# The Incidence, Intensity, and Risk Factors for Soil Transmissible Helminthes Infections among Waste Handlers in a Large Coastal Periurban Settlement in Southern Ghana

**DOI:** 10.1155/2021/5205793

**Published:** 2021-03-01

**Authors:** James-Paul Kretchy, Mawuli Dzodzomenyo, Irene Ayi, Duah Dwomoh, Kofi Agyabeng, Flemming Konradsen, Anders Dalsgaard

**Affiliations:** ^1^Department of Physician Assistantship Studies, School of Medicine and Health Sciences, Central University, Miotso, Accra, Ghana; ^2^Department of Biological, Environmental and Occupational Health Sciences, School of Public Health, College of Health Sciences, University of Ghana, Legon, Accra, Ghana; ^3^Department of Parasitology, Noguchi Memorial Institute for Medical Research, College of Health Sciences, University of Ghana, Legon, Accra, Ghana; ^4^Department of Biostatistics, School of Public Health, College of Health Sciences, University of Ghana, Legon, Accra, Ghana; ^5^Department of Public Health, Faculty of Health and Medical Sciences, University of Copenhagen, Copenhagen, Denmark; ^6^Department of Veterinary and Animal Sciences, Faculty of Health and Medical Sciences, University of Copenhagen, Copenhagen, Denmark; ^7^School of Chemical and Biomedical Engineering, Nanyang Technological University, Singapore

## Abstract

Soil-transmissible helminthes (STH) infections are among the most common sanitation-related public health problems in poor periurban settlements of tropical regions of low- and middle-income countries. In Ghana, research studies documenting the incidence rate, intensity, and occupational risk factors of STH infections among adults are scanty. A prospective cohort study of 261 waste handlers was conducted to investigate this. Stool samples were collected after 90 and 180 days of treatment with albendazole (400 mg per dose). The geometric mean intensity of STH among waste handlers after 180 days of treatment was 2.8 eggs/gram (light intensity), with an incidence rate of 1.5%. The proportion of waste handlers with light intensity STH infections was 4.8%. The odds of STH infection among female waste handlers were 80% lower when compared with male waste handlers (aOR = 0.2; 95% CI: 0.0–0.8). Waste handlers who used rubber gloves when working were 80% (aOR = 0.2: 95% CI: 0.2–1.9) protected from STH infections compared with those who did not use gloves. Infections with STH among the 261 waste handlers significantly correlated with the type of waste handling activities (LR *χ*^2^ = 15.3; *p*=0.033) with the highest proportion of infection found among transporters, 2 (40%). Waste handlers should receive periodic antihelminthic treatment, at least once every six months, practice adequate hand hygiene, and use suitable personal protective equipment during work.

## 1. Background

Soil-transmissible helminthes (STH) infections are among the most common global health problems associated with poor sanitation and lack of personal hygiene. Recent estimates suggest that nearly two billion people are infected globally [1–3], with over 80% of the disease burden found in tropical and subtropical regions of the world being associated with STHs [4, 5]. Several studies conducted in tropical regions of low- and middle-income countries (LMICs) have shown that residents in poor periurban communities have higher risks for acquiring STH infections, compared with high income urban settlements [6–10].

Poor periurban settlements are characterized by high levels of poverty, poor environmental hygiene, open defecation practices, and inadequate sanitation and waste management systems and thereby exposing both residents and waste handlers to the risks of STH infections [11]. The infective egg and larval stages of STH thrive in faecal-contaminated external environments, such as the soil. Thus, under favourable conditions and without the appropriate precautions of proper hand hygiene and wearing of personal protective equipment (PPE), waste handlers can be infected through faecal-oral transmission and ingestion of eggs or direct penetration of larvae through the skin [12–14].

Periodic use of antihelminthic medications is of key importance in controlling STH infections among at-risk populations in endemic countries [15, 16]. In Ghana, for example, albendazole (400 mg) and mebendazole (500 mg) are the medications used to treat STH infections [17–20]. These medications have undergone standard safety and efficacy testing and have been used extensively with only minor side effects [21]. Both medications are effective broad-spectrum antihelminthics, inexpensive, and are easily administered by trained nonmedical personnel. Albendazole (400 mg) is the preferred option for treatment of at-risk populations in prospective cohort studies because it is administered as a single oral dosage, which makes it easier to monitor by direct observation, unlike mebendazole which has to be taken once daily for three days [22–24]. The problem with preventive treatment using antihelminthic medications among at-risk populations, however, is the inability to prevent reinfection after a short period [25–28].

In Ghana, previous studies have focused on obtaining information about the prevalence, intensity, and associated risk factors of STH in children of school-going age [29, 30] and adult residents in urban areas [31, 32]. Knowledge on incidence rate, intensity, and risk factors of STH infection among people engaged in solid waste management, however, is scanty. Our study sought to investigate this and provide recommendations on how STH can be prevented and controlled among waste handlers in LMIC, such as Ghana.

## 2. Methods

### 2.1. Study Design

A prospective cohort study was conducted over a year between June, 2012 and August, 2013 involving workers occupied with different types of waste management in the study area. It involved questionnaire-guided interviews with heads or coordinators of waste management institutions and other relevant stakeholders to obtain permission and a transect walk to identify workers engaged in different waste handling activities in the study community. Subsequently, written informed consent was obtained from waste handlers to participate in the study. The participants were interviewed by the first author using a questionnaire on sociodemographic characteristics and types of PPE used. Antihelminthics were administered to workers under direct observation and stool samples collected after 90 days and 180 days of treatment. The stool samples were processed by the Kato-Katz technique [33] and examined by microscopy for ova of STH to determine incidence rate, prevalence, and intensity of infection. Data were analyzed as appropriate for statistical inferences.

### 2.2. Setting

The study was conducted in the poor coastal periurban community of Prampram, the administrative capital of the Ningo-Prampram district located in the southern part of Ghana. The district had a population of about 122,836 (GSS, 2013) and lies between latitude 5°45′ South and 6°05′ North and longitude 0°05′ East and 0°20′ West. The Prampram community has two major zones: lower Prampram (East and West), where people are predominantly engaged in marine fishing, and upper Prampram (Kley and Olowe), with mostly subsistence staple crop farmers. These areas did not have essential sanitation and waste management infrastructure, such as toilet facilities and proper solid waste disposal sites, at the time of the study. Over 50% of the residents were engaged in open defecation practices, creating multiple environmental and public health concerns associated with faecal contamination of the various solid wastes deposited in the external environment [34]. The prevailing climatic conditions in Prampram are hot and humid creating favourable survival conditions for the infective stages of STH to thrive.

### 2.3. Participants

#### 2.3.1. Description of Waste Handlers and Waste

Waste handlers were defined as sweepers, collectors, transporters, and disposers or those performing multiple waste handling activities, i.e., sweeping and collection, collection and disposal or sweeping, and collection and disposal. A detailed description of waste handler activities is provided in an earlier publication by our group [35]. The type of waste managed included solid waste mixed with fresh and decomposed human and animal faeces or effluents from domestic waste pipes and septic sludge from tanks emptied into open drains.

#### 2.3.2. Sampling Frame and Prestudy Observations

A mapping of waste management stakeholders in Prampram identified four main entities relevant to this study, namely, the Ningo-Prampram District Environmental and Sanitation Health Office (DESHO), the Local Government Assembly Office (LGAO), the District Waste Management Planning Office (DWMPO), and the Local Area Council for public toilet managers and waste transporters (LACPTMWT). Interviews were conducted with eight heads or coordinators of identified stakeholders who supervised waste handling activities in the district to establish a platform for the identification of waste handling activities and waste handlers and to also obtain consent for the study. The stakeholder interviews were eight in total, made up of two from DESHO, four members of LGAO, and one officer each from DWMPO and LACPTMWT. A transect walk was then undertaken after the interviews.

#### 2.3.3. Transect Walk and Observations

A transect walk through the periurban community to observe the study community for specific waste handling activities occurring at different sites (i.e., beaches, open dumping fields, and public toilet facilities) and to enumerate waste handlers was undertaken by the study team with the help of a local assistant. It also helped to get familiarized with the study area and obtain information about the state of waste management, access to hygiene and sanitation facilities, and the use of PPE among the waste handlers. The walk started from the outskirts of the community and gradually moved towards the centre [36]. After the transect walk and observation of the study area, 280 out of the total estimated 300 waste handlers gave informed consent and were recruited to participate in the study. Convenient sampling was used to select the waste handlers, based on the type of activities performed.

### 2.4. Variables

The main study outcome variable was soil-transmissible helminthes infections. The bodily surfaces exposed during waste handling, type of PPE, and type of waste management activities were recorded as exposure variables. Demographic and other variables, for example, age, sex, educational background, current monthly salary, type of waste management organisation, and number of work-years with waste management organisation were also considered as confounders/covariates with each other.

### 2.5. Data Sources and Measurements

#### 2.5.1. Questionnaire Administration

A questionnaire survey comprising 20 questions was administered by the first author to the 280 waste handlers to obtain information that included their sociodemographic characteristics, types of waste handling activities, exposure of bodily surfaces to the waste, and utilization of PPE. Questions were asked before beginning the day's work at locations where waste handling took place (i.e., beaches, open dumping fields, and public toilet facilities). Each respondent spent between 25 and 30 minutes to answer the itemized questions. The questionnaire was developed based on a previous work [37, 38] and modified within the context of the current study to reflect, for example, the different age range and average monthly salary of the waste handlers. The questionnaire was administered to the workers after a pretest was carried out at Dodowa, a nearby periurban setting with features similar to Prampram. The questionnaire was interpreted to waste handlers who could not read and write by trained bilingual speaking research assistants.

#### 2.5.2. Exclusion Criteria for Antihelminthic Administration

**Figure 1:**
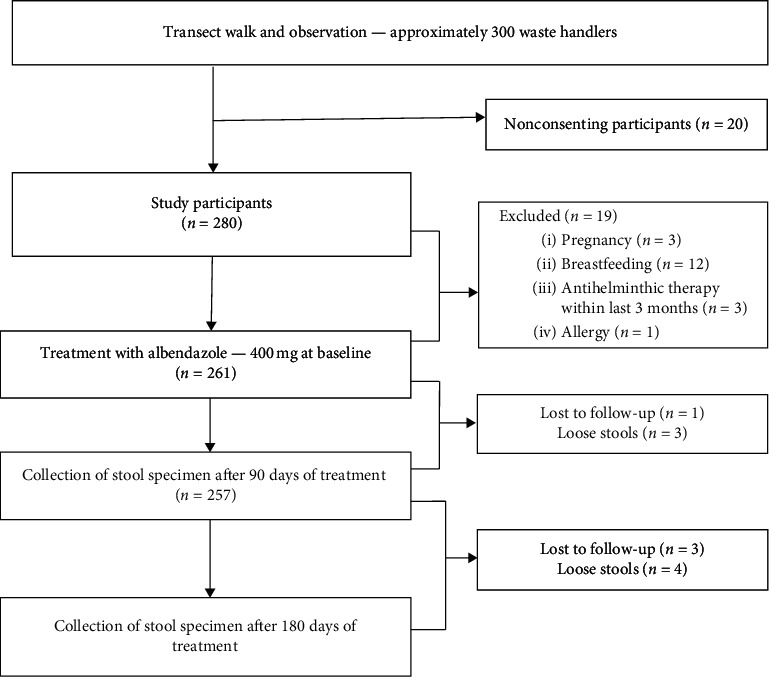
Flow diagram showing stage-by-stage recruitment of participants.

Nineteen waste handlers out of the 280 were excluded from taking antihelminthics due to pregnancy (*n* = 3), breastfeeding (*n* = 12), having taken an antihelminthic medication within the last three months (*n* = 3), and reported adverse reaction to an antihelminthic medication previously taken (*n* = 1) (Figure 1).

#### 2.5.3. Administration of Antihelminthics

According to Bonsu et al. [39], the prevalence rate of STH infections within the Ningo-Prampram community was 25.3%, which met the WHO criteria for mass treatment. Moreover, because of the presence of the conditions which favour transmission of STH such as high levels of poverty, poor environmental hygiene, open defecation practices, as well as inadequate sanitation and waste management systems, which were observed in Prampram, plus the occupational exposures to faecal-contaminated working environments in which the waste handlers worked [34], pretreatment prevalence rate of infections was not determined. The WHO recommends albendazole because it is effective in treating infected populations but harmless to uninfected ones [15]. After excluding the 19 waste handlers, the remaining 261 waste handlers were treated at baseline according to the WHO recommendations on antihelminthic treatment of high occupational risk groups living in endemic communities with a documented prevalence of over 20% [15]. Each person received a 400 mg single oral dose of albendazole (400 mg) using the directly observed treatment strategy [22, 40].

The treated waste handlers were then followed up in a prospective cohort, after 90 days and 180 days of treatment, to check their STH incidence rate and intensity of infections. The albendazole was administered by the first author after being trained by a health worker at the local Prampram Health Centre (PHC). There was a standby public health nurse at the PHC who was officially designated to receive any complaints of adverse reactions to the albendazole treatment. The anticipated complaints were upper gastrointestinal symptoms (e.g., epigastric or abdominal pain, nausea, and vomiting) and diarrhoea.

#### 2.5.4. Collection and Transportation of Stool Samples

Each waste handler produced an early morning stool which was collected in previously distributed labelled screw-capped stool specimen containers. The stools were placed in a cool box containing ice packs (to prevent decomposition of specimen and eggs from hatching) and transported within two hours to the Noguchi Memorial Institute for Medical Research (NMIMR) for processing and analysis. The stool samples were either processed and analyzed immediately or stored in a refrigerator at 4°C until ready to be processed within seven days [41].

#### 2.5.5. Processing and Analysis of Stool Samples by Kato-Katz Technique

Each stool sample was processed and analyzed using the Kato-Katz (cellophane faecal thick smear) procedure [33] with the following slight modifications. A small amount (about 1 g) of emulsified stool sample was placed on a hydrophobic paper in a biosafety cabinet. A piece of nylon sieve was pressed on top so that some of the stool specimens are sieved through the pores. A spatula was used to scrape the stool across the surface of the nylon sieve to fill the hole in the centre of a card template placed on a microscope slide. The hole in the template had been calibrated and found to accommodate approximately 42 mg of stool sample [42]. The excess stool was removed, and the template was carefully removed, and a cylinder of stool was left on the microscope slide. The stool sample on the microscope slide was covered with a cellophane strip presoaked with malachite green (prepared in glycerol). The slide was then inverted and firmly pressed against a leveled hard and smooth surface to spread the stool uniformly. The prepared slide was left in a dust-free area in the laboratory at room temperature (25–30°C) to clear for at least 30 min and examined under light microscope (×10 objective lens magnification).

The presence of any species of STH eggs identified was counted and recorded in a laboratory notebook. The ×40 objective lens was used to further confirm the morphology and size of STH eggs already identified with the ×10 objective. Two slides were prepared for each collected stool sample. The egg per gram (EPG) of faeces was calculated to obtain the intensity of STH infections by multiplying the number of individual eggs by the multiplication factor of 24 [43]. Only “formed” stool samples collected were suitable for processing by the Kato-Katz protocol. The “loose” stools were excluded from processing. Hardened stool samples were emulsified with a few drops of normal saline before processing.

After 90 days of treatment, 257 stool samples were processed owing to attrition (*n* = 1) and “loose” stool collected (*n* = 3), whilst 250 were collected after 180 days posttreatment due to attrition (*n* = 3) and “loose” stool collected (*n* = 4) (Figure 1). About 1 g of each processed stool sample was placed in Eppendorf tubes and stored at −80°C for molecular characterization of bacterial, viral, or protozoan agents in possible future studies.

### 2.6. Ethical Considerations

Ethical approval for the study protocols was obtained from the Institutional Review Board of the Dodowa Health Research Centre, Ghana Health Service, with review number (DHRC-IRB—STUDY No. 01/10/11), and the Ghana Health Service Ethical Review Committee (GHS-ERC—09/07/12) before commencement of data collection. An informed consent form, explaining the objectives of the study, risks and benefits, right to refuse, and confidentiality, was given and explained to the participants. Those who agreed were voluntarily recruited, after receiving their written consent. Study participants were assured of confidentiality and anonymity. Each participant was treated with respect.

### 2.7. Quality Control

Two slides were prepared per stool sample for identification and quantification of infective stages of STHs. Where present, the average number of STH eggs was used to estimate the intensity of infection. Parasite eggs on each positive slide were confirmed by a senior laboratory technician and microscopist. About 10% of all examined slides were randomly selected and reexamined under the microscope by the senior laboratory technician as quality control. The questionnaire was validated through a pretest in Dodowa, a similar study setting as Ningo-Prampram.

### 2.8. Data Management and Analysis

Observations from the transect walk were systematically recorded using observational guides and documented as field notes. The data from the questionnaire and EPG of STH were coded and entered into SPSS 17.0 for Windows 7 (SPSS, Inc., Chicago, IL.) and later imported into STATA MP Version 13 (STATA Corporation, College Station, USA) for statistical analysis. Descriptive analysis was used to summarize the data in the form of means, frequencies, and percentages. The likelihood ratio chi square test statistic was used to test the correlation between waste handling activities and STH infections.

A bivariate analysis was used to establish the risk factors associated with STH infections, and odds ratios were obtained at 95% confidence intervals (CI). Independent variables which had *p* values <0.05 at the bivariate analyses stage and were previously reported to be associated with STH infections were considered for inclusion in multivariate analysis. A multivariable logistic regression was performed to determine the joint effect of the risk factors and the incidence of STH infections. The egg counts for STHs were not normally distributed. Therefore, in determining the intensity of infections, the geometric mean was used to normalize the typical overdispersed distribution of EPG, as explained by Montresor [44]. The incidence rate of STH infection was estimated using the formula adopted by Brittney et al. (2013) with slight modification within the context of the current study as follows:

(1) $$\begin{array}{c} \frac{\text{Number of new cases of helminthes infections over the period}}{\text{Person}-\text{time at risk of helminthes infection over the follow}-\text{up period}}. \end{array}$$

The person-time at risk for STH infections was defined as [(*α*+*β*)/2] × Δ*t*, where *α* is the number of waste handlers at risk at the beginning of the time interval, *β* is the number of waste handlers at risk at the end of the time interval, and Δ*t* is the number of time units in the time interval.

## 3. Results

The transect walk and observations of waste management practices and general sanitary conditions revealed that waste handlers worked at different faecal polluted open spaces, including the beaches, around ponds and refuse containers, public toilet facilities, cemeteries, and in open drains receiving faecal sludge and domestic waste effluents. Waste handlers were engaged in sweeping, collection, transportation, and disposal of waste and a combination of two or more activities. An estimated majority (over 95%) of the workers had limited access to water, sanitation, and hygiene facilities during waste handling. For example, workers had to wash their hands with self-purchased sachet water without soap, before eating or after defecation during a working day. The results of the observation from the transect walk of the periurban community also showed that waste handlers used rudimentary waste handling equipment and technology such as brooms, wheel barrows, and shovels. Waste handlers were observed working in hot and humid conditions. Thus, noncompliance of the use of PPE may be linked to the discomfort when used during a hot workday.

### 3.1. Sociodemographic Characteristics and Waste Handling Practices

The sociodemographic characteristics of waste handlers who performed different activities are shown in Table 1. The 280 waste handlers (from the survey) were from five different waste management organizations operating within the study area, most of them being females (*n* = 211; 75.4%). The average mean age of the waste handlers was 42.7 ± 12.8 years. Approximately 217 (78.0%) of the study participants worked between three and four hours a day. 115 (41.1%) workers had no formal education, whilst only 13 (4.6%) had secondary education. A majority of waste handlers, 232 (82.9%), earned a monthly income of between 80 and 150 GH¢ (approximately USD 36–45), far below the average per capita monthly income of Ghana of 225 GH¢ (approximately USD 117) (GSS, 2013), which was the main source of income for most waste handlers.

The waste handlers worked with five different waste management institutions, anonymised as PC_1 (*n* = 155; 55.4%), private company 1 (workers in both lower and upper communities); AC (*n* = 20; 7.1%), area council (workers at public toilet facilities); PC_2 (*n* = 9; 3.2%), private company 2 (workers at upper communities); PC_3 (*n* = 51; 18.2%), private company 3 (workers at lower communities–beaches); and CVs (*n* = 45; 16.1%), community volunteers (workers in both lower and upper communities).

The proportions of waste handlers performing different activities were as follows: sweeping only, 51 (18.2%); disposal only, 18 (6.4%); collection only, 12 (4.3%); and transport only, 5 (1.8%). Workers who performed two or more activities included sweeping, collection, and disposal, 83 (30.0%); sweeping and collection, 69 (25.0%); collection and disposal, 36 (12.9%); and sweeping and disposal, 6 (2.1%).

The overall distribution of the use and type of PPE during work was as follows: Wellington boots, 173 (61.8%); gloves, 161 (57.5%); nose/mouth cover, 90 (32.1%); and overall apron, 204 (72.9%) (Table 1). The proportion of waste handlers with the highest use of gloves (51 (32%)) was those who performed sweeping, collection, and disposal activities, whilst none of the transporters used gloves during work (Table 1).

### 3.2. Incidence Rate and Intensity of STHs

The study revealed that the proportion of waste handlers that showed detection of STH after 180 days of treatment was 12 (4.8%), with *Trichuris trichiura* (*T. trichiura*) being the only detected STH species. The incidence rate of infection after 180 days of treatment was, however, 1.5%. No helminthes eggs were identified in stool sample of waste handlers after 90 days of treatment with albendazole. There was no STH infection recorded for waste handlers who engaged in only sweeping, only disposal, or who performed both activities a day (Table 2). Among the five (1.8%) waste handlers who transported waste everyday, two (representing 40%) had STH infections. Overall, the geometric mean intensity of STH was 2.8 ± 16.6 egg/gram after 180 days of treatment. According to the WHO classification (2006), intensity of *T. trichiura* between 1 and 999 egg per gram (epg) is light intensity, between 1000 and 9999 egg is moderate intensity, and >10,000 egg is heavy intensity. Even though the recorded intensity of STH infections after 180 days of treatment was light, waste handlers who only transported waste had the highest level of intensity (mean = 33.6 ± 44.1 SD) (Table 2). The proportion of light intensity STH infection rate among waste handlers was 4.8% after 180 days of treatment. In relation to moderate and high intensity STH infection, the prevalence was 0.0% in both categories (i.e., both after 90 and 180 days of treatment). A binary logistic regression analysis of factors associated with the presence of STH among waste handlers, using the likelihood ratio chi square test statistic, indicated a correlation between waste handling activities and STH infections (LR *χ*^2^ = 15.3, *p*=0.033), with the proportion of handlers categorized as transporters being the highest (*n* = 2; 40%).

### 3.3. Risk Factors Associated with the Presence of STH among Waste Handlers

The risk factor analysis showed that age, number of years of work, number of hours worked per day, waste handling activities, and surfaces of exposure did not correlate with STH infections after 180 days of treatment (*p* > 0.05) (Table 3). The sex of waste handlers and the use of gloves, as a PPE, were the only covariates associated with STH infections in the multivariable stage of analysis. The analysis showed that the odds of STH infections among female waste handlers were 80% (aOR = 0.2: 95% CI: 0.0–0.8; *p* = 0.009) less than the odds of infections among males. Similarly, waste handlers who used gloves were 80% (aOR = 0.2: 95% CI: 0.2–1.9; *p* = 0.003) less likely to acquire STH infections compared to those who did not use gloves (Table 3).

## 4. Discussion

Poor periurban communities of LMICs such as Ghana are confronted with limited waste management and sanitation infrastructure needed to serve the rapidly increasing populations. As a result, the hazards of faecal contamination of the solid waste stream are high [45, 46]. Waste handlers in such settings who directly handle solid waste along the waste management chain without ensuring safety measures such as hand hygiene and wearing of PPE are thus exposed to the risk of STH infections [35, 47].

Waste handlers in this study area had a significantly higher proportion of female engagement in sweeping compared with males, a finding which is similar to previous studies conducted by Agwu [48] in Nigeria and Kadfak (2011) in Ghana. Sweeping in waste handling is perceived by most traditional African communities as a predominantly female job. Indeed, certain minority tribes in Nigeria believe that a male is likely to lose sexual potency when touched by locally prepared brooms used for sweeping [49]. Our data found that the female waste handlers were less likely to have STH infections after 180 days of albendazole treatment compared with male workers (Table 3). This suggests that female waste handlers might have adopted better protective behaviour at work, by using safety working gear and adopting personal hygiene practices, or are involved in different types of waste handling activities (Table 3). Even though waste handlers were aware of the importance and timing of hand hygiene practices and use of sanitation facilities, observations at the work sites revealed that access to convenient hand hygiene facilities, i.e., water and soap for washing hands after work, after defecation and before eating, was lacking. The lack of these facilities in poor periurban communities of tropical countries may perpetuate the survival and transmission of STHs, particularly among occupational risk groups, such as waste handlers.

The data on socioeconomic status of the waste handlers suggest that the poverty levels in the periurban community were high, which might contribute to the incidence of STHs. We found that the highest income earned through waste handling was 150.0 GHS (approximately USD 29) a month, which was the main source of income for most workers. This was, however, far lower than the average monthly per capita income in Ghana, i.e., 225.0 GHS (approximately USD 44) (GSS, 2013). This confirms the perception that waste handlers in LMICs constitute one of the least paid workers, regardless of the reported high levels of health risks [50].

The type of waste handling activities correlated with STH infections, and among the five waste handlers who transported waste everyday, two (40%) had STHs, with the highest intensity of infection (Table 2). The waste handlers who used rubber gloves during work were better protected from acquiring STH infections compared with those who did not (Table 3). Yet, none of the waste transporters wore protective rubber gloves during work. Transporters were those driving waste-carrying tricycles loaded with faecal-contaminated solid waste in the rear caravan for disposal. It was therefore not surprising to detect the highest intensity among this category of waste handlers after 180 days of treatment. Working with faecal-contaminated solid waste in the studied poor periurban community without adequate protection of the hands was thus a risk factor to STH infections after 180 days of treatment. This finding has consolidated the knowledge on the importance of hands in the transmission of STHs and the need to wear PPE to prevent the direct exposure of bare hands to faecal-contaminated working environments [51]. It is well-known that faecal-contaminated hands can serve as transmission pathways to sanitation-related health problems such as diarrhoea in poor periurban settings, e.g., in Malaysia [52], and in other LMICs [53–56].

The low incidence rate and the light intensity of infection with STHs among waste handlers after 180 days of treatment with albendazole (400 mg) might be due to the treatment at baseline or the predominant adult population of waste handlers with an average age of 42 years, comparable with a study in Kenya where the average age was 38 years [57]. Research findings comparing children and adult populations have found that heavy intensity infections of STHs are typically seen in children rather than adults who were not treated at baseline [58, 59]. The highest incidence of STH in Ghana was reported among residents of an endemic community in Kintampo, along the middle belt of the country. The authors showed an incidence of 1.2% for the adult population and 0.8% for children [32, 60], which were less than the incidence rate we found for the waste handlers in Southern Ghana (i.e., 1.5%). The occupational exposures of waste handlers to faecal-contaminated solid waste in the poor periurban community of Prampram may account for this finding.

The most common health complaints following an infection from *T. trichura* include mainly intestinal manifestations, such as diarrhoea and abdominal pain [61], whilst heavy infections in adults have been associated with iron-deficiency anaemia [62]. Whilst our study did not investigate such health problems among the waste handlers, those infected could serve as reservoirs for the continued transmission of the infective stages to coworkers, their immediate family members, and the entire periurban community at large.

Public health interventions such as periodic treatment of waste handlers with albendazole (400 mg) to eliminate eggs of *T. trichiura* and other STHs at least once in every 180 days, wearing of rubber gloves to block transmission of infective stages from faecal-contaminated working environments, and use of hygiene facilities to reduce faecal-related biological agents on the hands are important measures in reducing the risk of acquisition and transmission of STH infections among waste handlers in poor periurban settlements [63]. Indeed, these measures are part of global strategies targeted at reducing risks to STH infections [64, 65]. Waste management institutions employing growing numbers of waste handlers must therefore adopt a holistic approach to prevent and control STHs among the workforce in the periurban settlement of Southern Ghana.

### 4.1. Limitations of the Study

Even though the current study was well conducted and the findings added valuable evidence to the incidence rate and intensity of STHs among waste handlers in Southern Ghana, there were few limitations. First, studies to assess the baseline prevalence of STH [66] and to measure the cure rate of albendazole (400 mg) two weeks posttreatment [67] would have added information on comparing STH infections before and after the intervention with albendazole. Second, the accuracy of the Kato-Katz technique in identifying waste handlers with STHs may be limited by day-to-day variation in egg excretion, and sensitivity is likely to be reduced when intensity of infections is low [68]. This potentially leads to lower than actually detected incidence of infection and mean infection intensities. Last, the study should have considered other nonwork specific risk factors to STH infections, for example, sanitation at home and use of footwear outside the working environment.

### 4.2. Strengths of the Study

In spite of the above limitations, the following are the strengths of the study: to the best of our knowledge, this is the first study to assess incidence rate and intensity of STH infections among waste handlers in Ghana to understand the extent of the challenge for appropriate public health interventions to be recommended. With a general scarcity of information on the risk factors to STH infections, the prospective cohort approach we used is the strength of our study and reflects what pertains in most poor periurban areas of LMICs.

## 5. Conclusion

The evidence from our study suggests that waste handlers in poor periurban settlements of LMICs such as the southern parts of Ghana are exposed to the hazards of faecal contamination of the solid waste, which predisposes them to STH infections. The incidence of light intensity STHs reported among waste handlers was higher than the national average, indicating higher exposures to risk factors associated with their occupation. Waste transporters were of the highest proportion to have STH infections. Female waste handlers plus those who used personal protective hand gloves during a working day were more protected from STH infections. The inadequate supply and use of PPE and limited access to sanitation and hygiene facilities among workers can aggravate STH infections. Therefore, efforts by waste management institutions at reducing the incidence, intensity, and risk factors to STHs to waste handlers must include improvement in sanitation/hygiene and waste management infrastructure. Waste handlers must ensure the efficient use of PPE to avoid direct contact of the bare hands with faecal-contaminated solid waste. Residents in the local communities must be aware of the dangers of outdoor defecation and avoid the practice. Recommendations for public health interventions to avoid infections with STH and other sanitation and hygiene-related diseases among waste handlers, their close contacts, and community members are encouraged.

## Figures and Tables

**Table 1:** Sociodemographic characteristics and type of personal protective equipment (PPE) by waste handlers performing different activities.

Sociodemographic characteristics/Type of PPE	Waste handling activities^a^, *n* (%)								Total (*n* = 280)
	Sweeping (*n* = 51)	Disposal (*n* = 18)	Collection (*n* = 12)	Transport (*n* = 5)	Sweeping and disposal (*n* = 6)	Sweeping and collection (*n* = 69)	Collection and disposal (*n* = 36)	Sweeping, collection, and disposal (*n* = 83)	
Sex									
Male	3 (4.4)	12 (17.4)	8 (11.6)	5 (7.3)	2 (2.9)	3 (4.4)	29 (42.0)	7 (10.1)	69 (24.6)
Female	48 (22.8)	6 (2.8)	4 (1.9)	0 (0.0)	4 (1.9)	66 (31.3)	7 (3.3)	76 (36.0)	211 (75.4)
Age in years									
<35	15 (16.7)	6 (6.7)	4 (4.4)	1 (1.1)	0 (0.0)	26 (28.9)	10 (11.1)	28 (31.1)	90 (32.1)
35 and above	36 (19.0)	12 (6.3)	8 (4.2)	4 (2.1)	6 (3.2)	43 (22.6)	26 (13.4)	55 (29.0)	190 (67.9)
Highest education level									
None	25 (21.7)	7 (6.1)	3 (2.6)	1 (0.9)	3 (2.6)	26 (22.6)	14 (12.2)	36 (31.3)	115 (46.0)
Primary	11 (14.1)	4 (5.1)	4 (5.1)	0 (0.0)	0 (0.0)	28 (35.9)	10 (12.8)	21 (26.9)	78 (27.9)
JHS	7 (23.3)	3 (10.0)	1 (3.3)	0 (0.0)	1 (3.3)	5 (16.7)	4 (13.3)	9 (30.0)	30 (10.7)
MSLC	6 (13.6)	3 (6.8)	3 (6.8)	3 (6.8)	2 (4.6)	8 (18.2)	5 (11.4)	14 (31.8)	44 (15.7)
Secondary	2 (15.4)	1 (8.0)	1 (7.7)	1 (7.7)	0 (0.0)	2 (15.4)	3 (23.1)	3 (23.1)	13 (4.6)
No. of years worked									
<1 year	16 (9.9)	7 (4.3)	9 (5.6)	1 (0.6)	2 (1.2)	54 (33.3)	22 (13.6)	51 (31.5)	162 (57.9)
1-2 years	17 (53.1)	3 (9.4)	0 (0.0)	1 (3.1)	0 (0.0)	2 (6.3)	1 (3.1)	8 (25.0)	32 (11.4)
3-4 years	17 (22.1)	7 (9.1)	3 (3.9)	2 (2.6)	4 (5.2)	11 (14.3)	12 (15.6)	21 (27.3)	77 (27.5)
5 or more years	1 (11.1)	1 (11.1)	0 (0.0)	1 (11.1)	0 (0.0)	2 (22.2)	1 (11.1)	3 (33.3)	9 (3.2)
Current monthly salary									
Less than ¢80.0 (<USD 36)	14 (32.6)	7 (16.3)	0 (0.0)	1 (2.3)	3 (7.0)	5 (11.6)	2 (4.7)	11 (25.6)	43 (15.4)
¢80–¢150 (USD 36–45)	37 (15.6)	11 (4.6)	12 (5.1)	4 (1.7)	3 (1.3)	64 (27.0)	34 (14.4)	72 (30.4)	237 (84.6)
Type of personal protective equipment									
Wellington boot	16 (9.3)	9 (5.2)	9 (5.2)	2 (1.2)	2 (1.2)	51 (29.5)	27 (15.6)	57 (33.0)	173 (61.8)
Gloves	25 (15.5)	9 (6.0)	11 (6.8)	0 (0.0)	2 (1.2)	39 (24.2)	24 (14.9)	51 (31.7)	161 (57.5)
Mouth/nose cover	15 (16.7)	5 (5.6)	3 (3.3)	3 (3.3)	0 (0.0)	23 (25.6)	15 (16.7)	26 (28.9)	90 (32.1)
Overall apron	19 (9.3)	10 (4.9)	12 (5.9)	3 (1.5)	2 (1.0)	62 (30.4)	30 (14.7)	66 (32.4)	204 (72.9)

^a^ *n* (%) represents frequency and row percentage. JHS, junior high school; MSLC, middle school leaving certificate.

**Table 2:** Intensity of STH infections, 90 and 180 days after albendazole treatment, categorized by the type of waste handling activities.

Type of activity	*n*	Intensity of helminthes infection (mean egg per gram (epg))		SD^a^ egg per gram/180 days posttreatment
		90 days/Posttreatment	180 days/Posttreatment	
Sweeping	51	—^b^	0 (0.0)	0.0
Disposal	18	—	0 (0.0)	0.0
Collection	12	—	1 (8.0)	20.8
Transportation	5	—	2 (33.6)	44.1
Sweeping and disposal	6	—	0 (0.0)	0.0
Sweeping and collection	69	—	5 (6.6)	28.0
Collection and disposal	36	—	2 (1.3)	5.5
Sweeping, collection, and disposal	83	—	2 (0.3)	2.6

^a^SD, standard deviation; ^b^no STH infections after 90 days of treatment.

**Table 3:** Sociodemographic and other risk factors associated with STH infections.

Sociodemographic/Risk factors	Helminthes infections 180 days after albendazole treatment		
	Frequency (%)	aOR^a^ (95% CI)	*p* value
Sex			
Male	6 (8.7)	1	
Female	6 (2.8)	0.2 (0.0–0.8)	0.009
Age in years			
<35	3 (3.3)	1	
35 and above	9 (4.7)	1.0 (0.9–1.1)	0.790
No. of years worked			
<1 year	6 (3.7)	1	
1-2 years	1 (3.1)	—^b^	0.319
3-4 years	4 (5.2)	3.2 (0.6–18.4)	
5 or more years	1 (11.1)	—	
Number of hours worked			
<1	1 (20.0)	1	
1-2	0 (0.0)	—	
3-4	10 (4.6)	—	0.691
>4	1 (4.0)	0.1 (0.0–7.0)	
Waste handling activity			
Sweeping	0 (0.0)	1	
Disposal	0 (0.0)	—	
Collection	1 (8.3)	8.3 (1.1–62.4)	0.963
Transportation	2 (40.0)	8.0 (0.08–87.0)	
Sweeping and disposal	0 (0.0)	—	
Sweeping and collection	5 (7.3)	8.1 (1.5–43.3)	
Collection and disposal	2 (5.6)	1.0 (0.1–8.0)	
Sweeping, collection, and disposal	2 (2.4)	—	
Type of protective working gear			
Wellington boot	10 (5.8)	—	—
Gloves	6 (3.7)	0.2 (0.2–1.9)	0.033
Mouth/nose cover	8 (8.9)	4.6 (2.0–16.6)	0.052
Overall apron	10 (4.9)	0.3 (0.0–3.2)	0.296
Exposure surfaces			
Mouth/nose	7 (6.0)	2.5 (0.6–9.7)	0.184
Hands	11 (4.6)	1.2 (0.3–5.8)	0.780
Leg/feet	6 (4.7)	1.8 (0.5–6.9)	0.397

% represents row percentage; ^a^aOR is the adjusted odds ratio estimate from a multivariable binary logistic regression analysis; ^b^parameter estimates were not possible since the number of workers that had the outcomes of interest were zero.

## Data Availability

The data used to support the findings of this study are included within the article and its supporting information files.

## Abbreviations

aOR: Adjusted odds ratio
GSS: Ghana Statistical Services
LR: Likelihood ratio
STH: Soil transmissible helminthes
SUSA: Sustainable sanitation
*T. trichiura*: *Trichuris trichiura*.

## Supplementary Materials

Supplementary Materials The questionnaire used for the survey.
