# Supplementary material for: The Incidence, Intensity, and Risk Factors for Soil Transmissible Helminthes Infections among Waste Handlers in a Large Coastal Periurban Settlement in Southern Ghana
Source: J Environ Public Health. 2021 Mar 1;2021:5205793. doi: 10.1155/2021/5205793 (PMC7949191; doi:10.1155/2021/5205793)
Supplement: Supplementary Materials — The questionnaire used for the survey. [file 5205793.f1.docx]

### **Questionnaire**

*Description: The purpose of this questionnaire was to identify demographic characteristics of waste handlers, exclusion criteria for administering anti-helminthic medication, types of activities undertaken, types of PPE used and parts of exposed bodily surfaces during work.*

| **Question number** | **Question** | **Response/s** |
| --- | --- | --- |
| **Section A**  General background information | | |
|  | Participants special ID # | ACPR….. ZLPR….  ZOPR….ARPR…. ASPR…. |
|  | Participants name |  |
|  | Participants Telephone number |  |
|  | Date of administering questionnaire | □□ □□ □□□□  dd mm yyyy |
|  | Consent has been read out to participant | Yes □  No □  If No, please read out. |
|  | Participant has given his/her consent | Yes □  No □  If Yes, continue with study, If No, please discontinue. |
| **Section B** Information for administering Albendazole (400mg) single oral dosage | | |
|  | What was your Date of Last Menstrual Period (LMP)? | If it is over a month do NOT administer drug |
|  | Are you breastfeeding? (For female waste handlers only) | Yes □  No □  If Yes, do NOT administer drug, If No, administer drug. |
|  | Do you have any known allergies/adverse reactions to de-wormers e.g. nausea, vomiting, abdominal pain? | Yes □  No □  If Yes, do NOT administer drug, If No, administer drug. |
|  | Have you taken any de-wormer within the past three months? | Yes □  No □  If Yes, do NOT administer drug, If No, administer drug. |
| **Section C**  Socio-demographic information | | |
|  | Sex (Indicate Male or Female as observed) | **□** Male  **□** Female |
|  | Age/Date of birth | Years…../□□ □□ □□□□  dd mm yyyy |
|  | Highest Level of Education | **□**None  **□** Primary  **□** MSLC  **□** Secondary  **□** Tertiary |
|  | Current Monthly Salary (State Current Salary Range in ¢GH) | **□** < ¢80  **□** ¢80 - ¢100  **□** Between ¢100 and ¢150 |
| **Section D**  Information about work | | |
|  | What waste management organization (WMO) do you work for? | **□** Zoom Lion  **□** ZOIL  **□** ACI  **□** Area Council  **□** Community volunteer |
|  | How long have you worked with this WMO? | **□** <1 year  **□** 1 – 2 years  **□** 3 – 4 years  **□** 5 years and above |
|  | What specific waste handling activity/ies do you perform? Tick all that apply to you. | **□** Collecting waste  **□** Disposing waste  **□** Sweeping waste  **□** Transporting waste |
|  | On the average, how many hours do you work per day? | **□** < 1hr  **□** 1-2 hrs  **□** 2-3 hrs  **□** 4-5 hrs  **□** Any other/Please specify_______ |
| **Section E**  Use of personal protective working gear and surfaces of exposure to waste material | | |
|  | What specific personal protective working gear/s do you use during waste handling? Please tick all that apply to you. | **□**Nose mask/mouth cover  **□** Over-all coat/apron  **□**Wellington boot  **□** Glove  **□** Any other/Please specify_______ |
|  | Which of your body surfaces has direct contact to waste material during waste handling? Please tick all that apply to you. | □ Hands  □ Leg/Feet  □ Nose/Mouth |
